# Dietary Sources, Stabilization, Health Benefits, and Industrial Application of Anthocyanins—A Review

**DOI:** 10.3390/foods13081227

**Published:** 2024-04-17

**Authors:** Ramesh Kumar Saini, Mohammad Imtiyaj Khan, Xiaomin Shang, Vikas Kumar, Varsha Kumari, Amit Kesarwani, Eun-Young Ko

**Affiliations:** 1 School of Health Sciences and Technology, UPES, Dehradun 248007, Uttarakhand, India; rameshkumar.saini@ddn.upes.ac.in; 2 Biochemistry and Molecular Biology Lab, Department of Biotechnology, Gauhati University, Guwahati 781014, Assam, India; imtiyaj@gauhati.ac.in; 3 Jilin Provincial Key Laboratory of Nutrition and Functional Food, Jilin University, Changchun 130062, China; xmshang@jlu.edu.cn; 4 Department of Food Science and Technology, Punjab Agricultural University, Ludhiana 141004, Punjab, India; vikaschopra@pau.edu; 5 Department of Plant Breeding and Genetics, Sri Karan Narendra Agriculture University, Jobner, Jaipur 302001, Rajasthan, India; varshagpb@gmail.com; 6 Department of Agronomy, College of Agriculture, Govind Ballabh Pant University of Agriculture and Technology, Pantnagar 263145, Uttarakhand, India; getkesar@gmail.com; 7 Department of Food Science and Biotechnology of Animal Resources, Konkuk University, Seoul 05029, Republic of Korea

**Keywords:** flavonoids, berries, cyanidin, acylation, co-pigmentation, microencapsulation, food colorant, active and intelligent packaging

## Abstract

Natural phytochemicals are well known to protect against numerous metabolic disorders. Anthocyanins are vacuolar pigments belonging to the parent class of flavonoids. They are well known for their potent antioxidant and gut microbiome-modulating properties, primarily responsible for minimizing the risk of cardiovascular diseases, diabetes, obesity, neurodegenerative diseases, cancer, and several other diseases associated with metabolic syndromes. Berries are the primary source of anthocyanin in the diet. The color and stability of anthocyanins are substantially influenced by external environmental conditions, constraining their applications in foods. Furthermore, the significantly low bioavailability of anthocyanins greatly diminishes the extent of the actual health benefits linked to these bioactive compounds. Multiple strategies have been successfully developed and utilized to enhance the stability and bioavailability of anthocyanins. This review provides a comprehensive view of the recent advancements in chemistry, biosynthesis, dietary sources, stabilization, bioavailability, industrial applications, and health benefits of anthocyanins. Finally, we summarize the prospects and challenges of applications of anthocyanin in foods.

## 1. Introduction

Anthocyanins are water-soluble vacuolar pigments belonging to the parent class of flavonoids [1]. They are widely distributed in fruits, flowers, and grains and are responsible for intense red, orange, blue, and purple colors [1,2]. The function of anthocyanins in plants is to prevent the production of damaging free radicals and shield leaves, flowers, and fruits from ultraviolet light [3,4].

Approximately, over 600 unique anthocyanins have been identified in nature. Anthocyanins are synthesized through the phenylpropane pathway in the cytoplasm and accumulate in the vacuoles of plant cells. Individual plant species possess a unique genetically predetermined pattern of anthocyanin accumulations. However, their biosynthesis and accumulation are significantly influenced in response to various biotic and abiotic factors [5,6,7,8].

Anthocyanins are abundantly found in fresh fruits such as berries, concord grapes, and pomegranates, vegetables such as eggplant, black/purple carrot, red cabbage, violet cauliflower, purple sweet potato, and colored gains [9]. Consumption of these anthocyanin-rich fruits, vegetables, grains, and their products is linked to various health benefits [10,11,12,13,14]. These effects are primarily attributed to the antioxidant properties exhibited by anthocyanins [1,15,16]. Moreover, recent studies revealed that anthocyanin has prebiotic activity, which helps to keep the body healthy by promoting the proliferation of beneficial gut microbiota while inhibiting the growth of harmful bacteria [17,18,19,20,21]. Moreover, anthocyanin possesses potent antiviral properties against pathogens [22].

Anthocyanins are prone to degradation by various external environmental conditions, including temperature, pH, oxygen, light, and enzymes [23,24,25]. Various techniques, including microencapsulation, have been innovated to improve thermal and storage stability, solubility, and bioavailability [26,27,28,29].

Anthocyanin-rich aqueous extracts used as food dyes are considered safe [14,30]. Moreover, owing to the health benefits of consuming natural anthocyanins, they are widely used as a natural pigment in various foods, including candies, jams, jelly, and fresh sausages, as well as in beverages such as yogurts and juices. Also, anthocyanins-based composite films are widely investigated to extend the shelf-life of food and monitor the freshness of protein-rich food products in real time [31,32,33,34]. Because of the pH-sensitive properties of anthocyanins, the pH changes are sensed by anthocyanin-based films and change their color, enabling visual monitoring of the freshness of protein-rich food [35,36]. 

Numerous exceptional reviews focusing on anthocyanins have been recently published, centering primarily on their structural diversity in land plants [2], biosynthesis and regulation during biotic and abiotic stress [6,37,38], stabilization approaches, including nano/microencapsulation [39,40,41,42], health benefits [1,10,14,20,43,44,45,46], and preparations and application of anthocyanins-based composite films [34]. However, despite these individual reviews, a comprehensive review integrating all these aspects of anthocyanins is lacking. Thus, this review aims to provide a holistic view of recent advancements in diverse topics, encompassing chemistry, biosynthesis, dietary sources, stabilization, bioavailability, industrial applications, and health benefits of anthocyanins. Notably, this review emphasizes the applications of anthocyanins in food and industries while highlighting their health benefits, as observed in pre-clinical and clinical studies.

## 2. Literature Search Methodology

Available electronic databases, especially Google Scholar, PubMed, and Web of Science, were searched for studies dealing with dietary sources, stabilization, health benefits (in vitro, in vivo, and clinical), and industrial application of anthocyanins. The primary search keywords were (1) anthocyanin (title) and health (topic) and (2) anthocyanin (title) and bioactive (topic). The other keywords were the following: (1) anthocyanin (title) and berries (title); (2) anthocyanin (title) and biosynthesis (title); (3) anthocyanin (title) and acylation (topic); (4) anthocyanin (title) co-pigmentation (topic); (5) anthocyanin (title) and encapsulation (title); (6) anthocyanin (title) and bioavailability (title); (7) anthocyanin (title) and food colorant (topic); and (8) anthocyanin (title) and intelligent packaging (topic). The relevant 376 articles downloaded were published mainly between 2018 and 2024. A total of 171 articles were discussed in this review.

## 3. Structure and Properties of Anthocyanins

**Figure 1:**
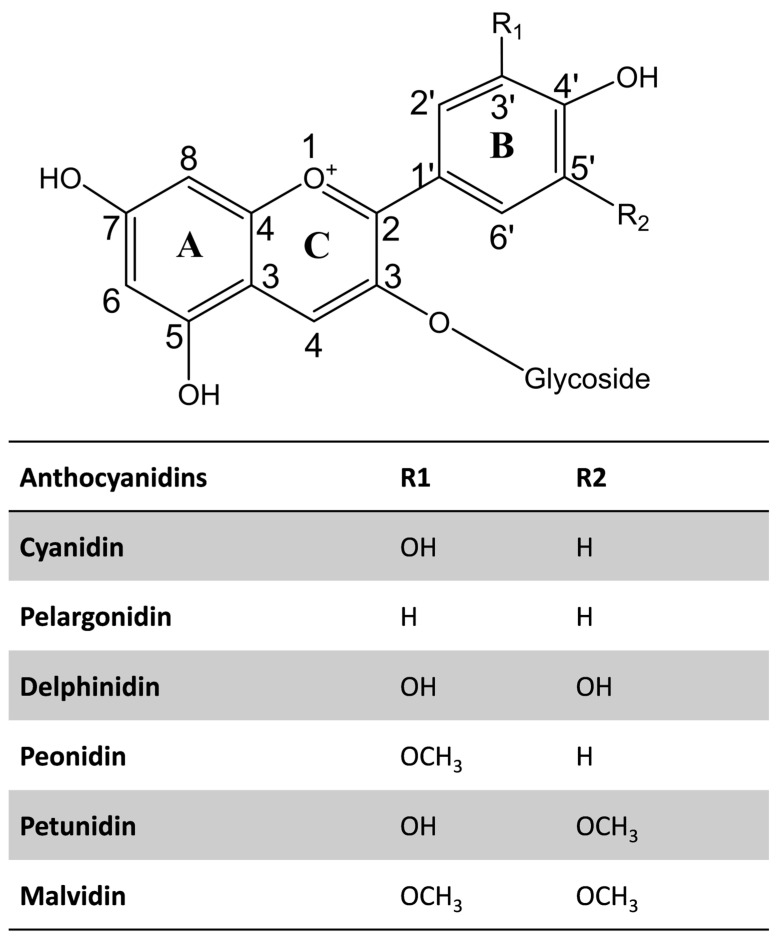
The structure of commonly occurring anthocyanidins.

Structurally, anthocyanins belong to the flavonoid family and consist of a typical C6–C3–C6 basic skeleton, composed of two aromatic rings (A and B) joined by a six-membered heterocyclic ring (C) containing oxygen, which is further conjugated with a sugar moiety (Figure 1). The variations in the B-ring substituents, such as hydroxyl and methoxy groups, the nature and position of the sugar attachments, and the degree of acylation, contribute to the structural diversity and stability of anthocyanins. Saccharides, including glucose, galactose, rhamnose, arabinose, rutinose, xylose, sophorose, sambubiose, and/or glucorutinose are attached to the aglycone of anthocyanins [2]. Also, anthocyanins can be acylated with aliphatic and aromatic acids, including caffeic, *p*-coumaric, *p*-hydroxybenzoic, gallic, succinic, oxalic, malonic, ferulic, and/or sinapic acids [2,47]. 

The chemical diversity of anthocyanin in the plant kingdom is reviewed elsewhere [2]. The most abundant anthocyanidins include cyanidin, delphinidin, malvidin, peonidin, pelargonidin, and petunidin (Figure 1). Interestingly, cyanidin has the highest diversity among them. Among the 434 commonly occurring anthocyanins identified in 639 plant species, cyanidin accounts for 33% of the identified anthocyanins [2]. Moreover, 3-*O*-glucosides or 3,5-*O*-diglucosides of anthocyanidins most commonly occur in plants.

## 4. Anthocyanins Are Sensitive to the External Environment

Anthocyanins, being highly susceptible to degradation, can undergo chemical breakdown due to exposure to a variety of external environmental factors, notably under higher temperatures, alkaline pH levels, oxygen presence, light exposure, and enzymatic activity of polyphenol oxidase [23,24,25]. In addition, oxidants (e.g., H_2_O_2_), reducing agents (e.g., Na_2_SO_3_), metal ions of Mg^2+^, Cu^2+^, and Al^3+^, and ascorbic acid have detrimental effects on anthocyanins [48]. The mechanism of ascorbic acid-mediated anthocyanin degradation is not entirely elucidated. However, it is believed that the condensation reaction between ascorbic acid and carbon-4 (most susceptible to electrophilic attack) of the anthocyanin [49] or the free radicals generated by oxidation of ascorbic acid can breakdown the flavylium core of the anthocyanins [48]. Extensive bleaching was observed in cyanidin-3-galactoside of chokeberry extract when 1 mg/mL ascorbic was added, with a significant decrease in half-lives from 22.8 to just 0.3 days.

In general, glycosylated anthocyanin forms are more stable than anthocyanidins [23]. Thus, to minimize the degradation of anthocyanins, packaging material providing an air barrier to control the storage atmosphere with a vacuumed packaging process is advisable [23]. Moreover, the anthocyanins are stored at a low temperature with a minimum exposure to light.

Anthocyanins are better protected in acidic environments, whereas neutral and alkaline conditions promote the degradation of anthocyanin [25]. For instance, the stability of anthocyanins from purple sweet potato extract investigated at 90 °C showed half-lives of 10.27, 12.42, and 4.66 h at pH 3.0, 5.0, and 7.0, respectively [25].

Crude extracts exhibit higher thermal stability of anthocyanins compared to purified ones, probably due to the occurrence of intra and inter-molecular co-pigmentation reactions. Additionally, interactions with proteins and polysaccharides of their own food matrices during processing influence anthocyanin degradation, leading to a loss of anthocyanin content ranging from 28% to 80% [50]. The average half-life of anthocyanin degradation at 60 °C is 19.7 h for fruits and 22.28 h for vegetables, and this duration diminishes significantly with higher temperatures [50].

**Figure 2:**
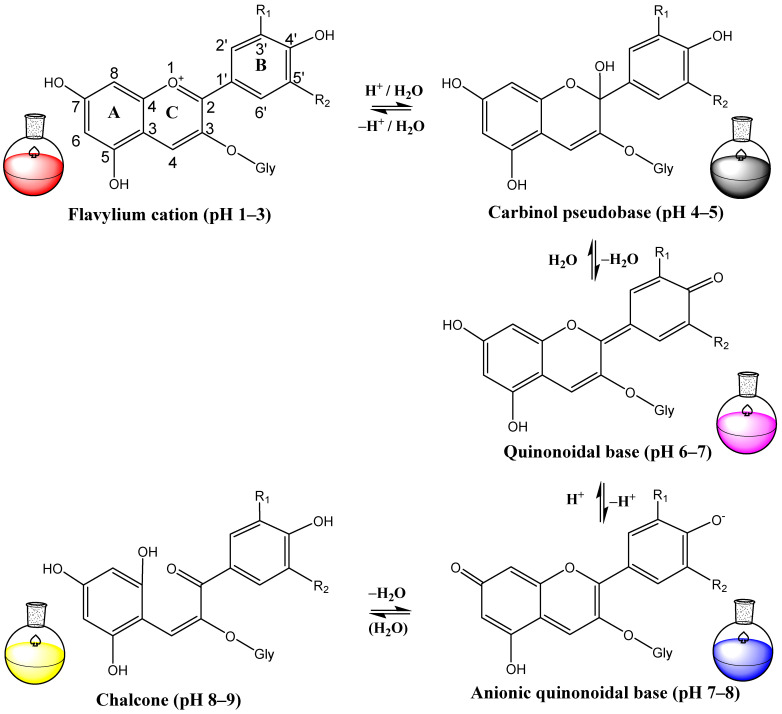
pH-dependent changes in chemical structures of anthocyanin.

In acidic solutions with pH values ranging from 1 to 3, anthocyanins exist as flavylium cations, characterized by oxonium-charged oxygen. However, in an alkaline solution, they can donate electrons and transform into quinonoidal bases, with subsequent loss of coloration [31,51,52]. Subsequently, the C2-O bond of the C-ring of anthocyanin is cleaved, producing a colorless chalcone compound (Figure 2).

## 5. Anthocyanins Are Potent Antioxidants 

Oxidative stress, a consequence of an impaired balance between the antioxidant mechanism and oxidative conditions, is considered the main contributing factor in developing chronic diseases, cardiovascular diseases, diabetes, obesity, neurodegenerative diseases, and cancer [53]. During the normal metabolism of the body, reactive oxygen species (ROS), including hydroxyl radical (HO^•^), superoxide radical anion (O2^•−^), singlet oxygen (^1^O_2_), and hydrogen peroxide (H_2_O_2_), are produced [53]. In vitro studies suggest that anthocyanins exhibit potent antioxidant properties primarily due to their capacity to contribute hydrogen atoms or electrons to neutralize ROS [54]. Thus, the antioxidant properties make anthocyanins promising candidates for preventing oxidative stress-related diseases and promoting human health.

The structure–activity relationship of anthocyanin demonstrated that hydroxylation at positions C3′ and C5′ of the B-ring enhances the capacity for hydrogen donation, indicating that the B-ring primarily contributes to electron donation [55]. Studies indicated that delphinidin exhibits the highest antioxidant activity among the six anthocyanidins, probably due to the presence of three hydroxyl groups at positions C3′, C4′, and C5′ of the B-ring [56]. Furthermore, the antioxidant activity of anthocyanins is increased through acylation, while glycosylation decreases it [57], likely due to the heightened chemical reactivity resulting from these modifications.

The antioxidant potential of anthocyanin is highlighted in Table 1. A study on fruits of 61 *Lonicera caerulea* L. genotypes rich in anthocyanins (158.4–1751.4 mg/100 g fresh weight (FW)) suggested cyanidin-3,5-diglucoside (containing 1.6–9.8% of total anthocyanin) demonstrate the highest ROS scavenging activities, compared to other dominantly occurring anthocyanins, e.g., cyanidin-3-*O*-glucoside, which represent 80.1–91.3% of the total anthocyanins in these genotypes.

In our study, anthocyanin-rich red lettuce cultivars showed nearly 5 times more ABTS (2,2′-azino-bis-(3-ethylbenzothiazoline-6-sulphonic acid)) and DPPH (2,2-diphenyl-1-picrylhydrazyl) radical scavenging activities, compared to green lettuce cultivars, which lack anthocyanin [58]. We observed a similar observation from perilla (*Perilla frutescens* (L.) Britt.) foliage, where anthocyanin-rich green/red perilla showed 3 times more DPPH radical scavenging activities, compared to green perilla, which lacks anthocyanins [59].

Due to the potent antioxidant activities, anthocyanin-rich black carrot extract has been shown to protect against the oxidation of polyunsaturated fatty acids (PUFAs) in liposomes [60], which are widely used as a delivery method for antimicrobials, vitamins, and bioactive compounds. Currently, synthetic antioxidants are widely utilized to prevent the oxidation of PUFAs (associated with phospholipids of liposomes) [60].

**Table 1:** Antioxidant potential of anthocyanins.

Plant/Food	Anthocyanins Studied	Method Used for Determining the Antioxidant Activity	Key Results	References
61 genotypes of *Lonicera caerulea* L. fruits	Nine anthocyanins including, cyanidin-3,5-diglucoside, cyanidin-3-*O*-rutinoside, cyanidin-3-*O*-glucoside, and peonidin-3-*O*-glucoside	Superoxide anion and hydroxyl radical scavenging activities	Cyanidin-3,5-diglucoside demonstrate the highest ROS scavenging activities	[61]
Green/red and red lettuce cultivars	Cyanidin (87.9–3656.9 µg/g DW)	ABTS^•+^ and DPPH^•^ radical scavenging activities	The cyanidin contents and the highest DPPH and ABTS activity were recorded in “Caesar red” and “Jinbballolla” cultivars	[58]
Perilla (*Perilla frutescens* (L.) Britt.) foliage	Cyanidin 3-*O*-(6-*O*-p-coumaroyl) glucoside-5-*O*-malonyl glucoside (malonyl-shisonin)	ABTS^•+^ and DPPH^•^ radical scavenging activities	Green/red perilla showed 3-times more DPPH radical scavenging activities compared to green perilla, which lacks anthocyanins	[59]
Purple wheat products (flour and bran)	Cyanidin-3-glucoside, cyanidin-3- rutinoside, cyanidin-3-(6-malonyl glucoside), peonidin-3-(6-malonylglucoside), and peonidin-3-glucoside	ABTS^•+^ and DPPH^•^ radical scavenging and ORAC assay	The anthocyanin contents correlated with the antioxidant activity	[62]
Blueberry anthocyanins	14 anthocyanins including malvidin-3-*O*-glucoside (2.36 mg/g), malvidin-3-*O*-galactoside, and petunidin-3-Oglucoside	ABTS^•+^ and DPPH^•^ radical scavenging, FRAP, reducing power and superoxide anion radical scavenging activity	Anthhocynins showed nearly two times higher antioxidant activities than the standard ascorbic acid.	[63]
*Hibiscus sabdariffa* calyx	Cyanidin-3-glucoside, delphinidin-3- Glucoside, delphinidin-3-sambubioside, and cyanidin-3-sambubioside	DPPH^•^ scavenging activity	The antioxidant activity correlated positively with anthocyanins (r = 0.48)	[64]
Red raspberry anthocyanin microcapsules	Not analyzed	DPPH^•^ radical scavenging activity	Anthocyanin microcapsules prepared with gum Arabic showed higher antioxidant activity than that of maltodextrin microcapsules. The antioxidant activity increased gradually with increased anthocyanin contents in the microcapsules	[65]
Raspberry	Cyanin-3-*O*-glusoside	ABTS^•+^ and DPPH^•^ radical scavenging and ORAC assay	Antioxidant activity of nonacylated raspberry anthocyanins was similar to that of acylated anthocyanins, as well as ascorbic acid	[66]

DW: dry weight; ABTS^•+^: 2,2′-azino-bis-(3-ethylbenzothiazoline-6-sulphonic acid); DPPH^•^: 2,2-diphenyl-1-picrylhydrazyl; ORAC: oxygen radical absorbance capacity; FRAP: ferric reducing antioxidant power.

In addition to its antioxidant properties, anthocyanins possess potent antimicrobial properties [67]. Chinese wild blueberry anthocyanins at the concentration of 0.53 mg/mL have shown cytotoxic properties against various foodborne pathogens, including *Vibrio parahaemolyticus* (most susceptible to anthocyanin), *Staphylococcus aureus*, *Listeria monocytogenes*, and *Salmonella enteritidis*, by increased cell membrane permeabilization and leakage of nucleic acid and proteins, inhibition of TCA cycle and protein synthesis, and reducing the energy transfer [67].

## 6. Biosynthesis and Regulation

Anthocyanins are synthesized through the phenylpropane pathway in the cytoplasm and accumulate in the vacuoles of plant cells. Their biosynthesis is largely controlled by genetic factors [6]. However, various environmental factors can also regulate their biosynthesis, including low temperature (e.g., during autumn), light, ultraviolet radiation, and drought [5,6,7,8]. They can protect cells from damage caused by these stress conditions and play an important role in adaptation under such stressed conditions [5,37]. Specifically, the role of light and temperature on anthocyanin accumulation has been well investigated [68]. The plant growth regulators, including abscisic acid, ethylene, jasmonate, Brassinosteroid, auxin, cytokinins, and gibberellin, are known to influence the anthocyanin accumulation in plants [69,70,71,72]. Moreover, nitrogen and phosphate deficiencies, regulated deficit irrigation, and exogenous sugar application also lead to enhanced biosynthesis of anthocyanin [6,73,74]. Also, the biosynthesis of anthocyanin is triggered during the ripening of fruits [68].

**Figure 3:**
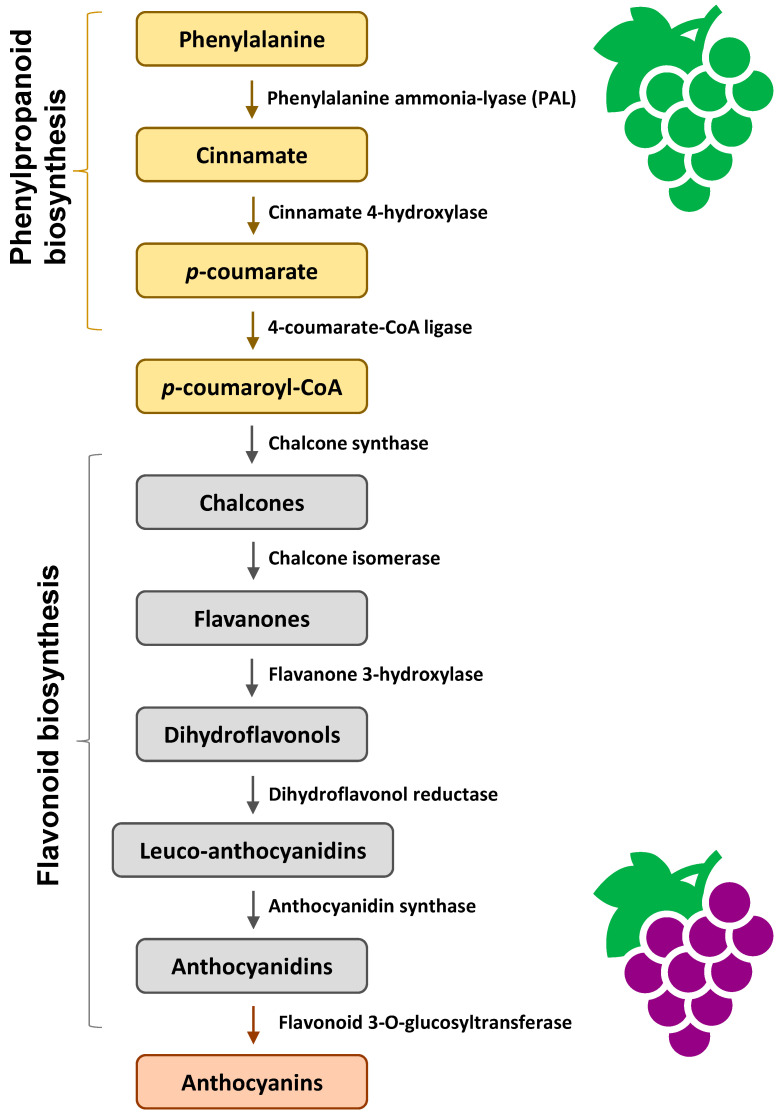
An outline of anthocyanin biosynthesis pathway [38].

Anthocyanin biosynthesis is facilitated by a series of enzymes encoded by structural genes (Figure 3). In the early stage of phenylpropanoid biosynthesis, phenylalanine is converted to *p*-coumaroyl-CoA, catalyzed by a series of enzymes. In the middle stage of flavonoid biosynthesis, *p*-coumaroyl-CoA is converted to dihydroflavonols. Finally, anthocyanidins are formed. The anthocyanidins can be modified by methylation, glycosylation, and acylation to form stable anthocyanins [38].

The expression of anthocyanin biosynthesis genes is mainly controlled by the MYB-bHLH-WD40 (MBW) complex, composed of R_2_R_3_MYB (RYM), basic helix–loop–helix (bHLH), and WD_40_ repeats [68,75]. During abiotic stress, an increased amount of ROS is produced, which acts as a signaling mediator to trigger the upregulation of anthocyanin biosynthesis pathway genes via interactions with regulatory transcription factors (e.g., MYB-bHLH-WD40 protein complex). These anthocyanins are utilized to detoxify the ROS and maintain cellular osmotic balance, thereby increasing abiotic stress tolerance and helping plants sustain adverse conditions [37].

In contrast to this highly conserved core activation complex, several anthocyanin repressors have been identified in the plants, which act either as repressing the transcription of the MBW complex or destabilizing it via protein–protein interactions [38].

A recent study suggested that the accumulation of anthocyanins in the young leaves of tea (*Camellia sinensis* L.) cultivars, specifically Zifuxing 1, is primarily influenced by the plant’s response to high light intensity [76]. Authors proposed that exposure to high light intensity induced ROS, subsequently triggering the synthesis of abscisic acid, which interacts with MYB transcription factors, resulting in enhanced anthocyanins biosynthesis and the purplish coloration observed in the young leaves. Thus, an alternative to the high light intensity, the external application of ABA can promote anthocyanin accumulation in purple-leaf cultivars, as such cultivars are gaining more interest in the production and consumption of tea due to the health-beneficial effects of anthocyanins. 

Moreover, 5-Aminolevulinic acid, an essential biosynthetic precursor of tetrapyrrole compounds (chlorophyll, heme, and B12), promotes anthocyanin accumulation in many plant species, including apple [77].

## 7. Dietary Source and Intake

Fresh fruits such as berries, apples, figs, plums, concord grapes, and pomegranates, vegetables such as eggplant, black/purple carrot, red cabbage, violet cauliflower, purple sweet potato, and colored gains are the major dietary sources of anthocyanins. Among berries, chokeberry (*Aronia* sp.), blueberry, blackberry, raspberry, cherry, mulberry, strawberry, Chinese bayberry (*Myrica rubra* Siebold & Zucc.), *Lonicera edulis*, and *lycium Chinensis* are the richest source of anthocyanins [9]. The amount of anthocyanins in berries varies from 10 (wild strawberry) to 772.4 mg/100 g FW (bilberry) [1]. Moreover, comparable levels of anthocyanins were found in the commercial jams prepared from raspberry, blackcurrant, blueberry, blackberry, and cranberry, suggesting that commercially available berry jams can also serve as a significant source of anthocyanins [78].

Individual plant species possess a unique genetically predetermined pattern of anthocyanin accumulations. Most commercial anthocyanin sources, such as berries, contain nonacylated anthocyanins [79]. Acylated anthocyanins are abundant in black goji berry, purple corn, purple-fleshed potato, purple sweet potato, red radish, purple and black carrot, red cabbage, and red radish, as reviewed elsewhere [79].

Cyanidin 3,5-diglucoside and cyanidin 3-diglucoside-5-glucoside derivatives acylated with caffeic, p-coumaric, p-hydroxybenzic, oxalic, malonic, ferulic, and/or sinapic acids are the major anthocyanins identified in *Brassica* vegetables, including violet cauliflower and red cabbage [47].

Cyanidin-3-*O*-glucoside, followed by cyanidin-3-*O*-rutinoside, are the major anthocyanins in mulberry (*Morus* sp.) fruits. Among the 12 genotypes of mulberry fruits cultivated in the Republic of Korea, *Morus Alba* L. *cv*. Iksu and *Morus Microphylla* Buckl. *cv*. Shimgang showed the highest cyanidin-3-*O*-glucoside content of 19.51 and 18.92 mg/g dry weight (DW), respectively [80].

In blueberries, anthocyanins constitute 60–70% of the total polyphenolic compounds, with the total anthocyanins containing 58.5–255.6 mg/100g FW [81]. Malvidin glycosides were the major anthocyanins in 20 genotypes of highbush blueberry genotypes grown in British Columbia, Canada [81].

Among vegetables, red lettuce cultivars are a rich source of anthocyanin [58,82]. Cyanidin 3-*O*-(6′-*O*-malonyl) glucoside is the most dominant anthocyanin (97% of total anthocyanins), with a trace amount of peonidin-3-*O*-glucoside, present in most commercial and traditional lettuce (*Lactuca sativa* L.) and their wild relatives [82]. Among 30 *Lactuca* accessions investigated for the anthocyanin contents, the highest anthocyanin of 127.28 mg/100g DW was recorded in commercial red lettuce (‘Likarix’). In our study, 155.8 (*cv*. Super Caesar red)–365.6 mg/100 g DW (*cv*. Caesar red) of cyanidin was recorded in red lettuce harvested at the baby-leaf stage [58].

Among cereals, colored maize, rice, and wheat grains are the rich source of anthocyanin [83,84,85]. Cyanidin-3-*O*-glucoside (1.2–110.6 mg/100 g) and peonidin-3-*O*-glucoside (0.35–31.1 mg/100 g) are the dominant anthocyanins in purple/back rice varieties [83,86]. Similarly, cyanidin-3-glucoside is dominantly found in purple wheat [62]. The seed coat of black soybean is also a rich source of anthocyanins, especially cyanidin-3-*O*-glucoside and cyanidin-3-*O*-galactoside [87].

In addition to the land plants, anthocyanin can be successfully produced in vitro, utilizing microbial and plant cell or tissue culture [88,89]. However, these approaches have mostly been tested at the lab and pilot scales and are not a significant source of industrial production of anthocyanin.

Due to the high anthocyanin content, berries are the primary source of anthocyanin, accounting for 39 and 43% of total anthocyanin intake in the United States of America (USA) and Europe, respectively [44], while red wine, vegetables, and other fruits contribute 22–18, 19, and 9% of total anthocyanin intake in the USA and Europe [44]. Red wine contains up to 2000 mg/L of anthocyanin [90].

In Europe, the anthocyanin intake ranges from 18.4 (Spain) to 44.1 mg/day (Italy) in women and 19.8 (the Netherlands) to 64.9 mg/day (Italy) in men [44]. In Asian countries, Australia, and the USA, the average intake is 37, 24.2, and 12.5 mg/day, respectively [44]. Being a non-essential nutrient, the recommended daily intake is not established. According to the European Food Safety Authority (EFSA), the currently available toxicological database was inadequate to establish a numerically acceptable daily intake (ADI) for anthocyanins [14,30]. However, 50 mg/day of daily anthocyanin intake in China is recommended [44]. Also, according to an exposure estimate, for an average adult weight of 70 kg, anthocyanin intake of 49–133 mg/day could be well-tolerated [14].

## 8. Stabilization of Anthocyanins

In acidic solutions, anthocyanins exist as flavylium cations, which provide intense red coloration. However, in an alkaline water solution, they can donate electrons and transform into quinonoidal bases, with subsequent loss of coloration [31,51,52]. Therefore, preserving the flavylium cation against nucleophilic attack by water and oxidants is considered an effective strategy to protect anthocyanins [91].

Several strategies have been developed to protect anthocyanins from adverse environmental conditions, which include the following: (1) changing its structure by chemical structural modification (glycosylation, acylation) [28]; (2) combining them with biological macromolecules such as proteins and polysaccharides to form stable complexes [26,27]; (3) micro-/nano-encapsulation to provide a physical barrier that shields the anthocyanins from environmental factors; and (4) co-pigmentation [91]. Moreover, these strategies help minimize degradation and preserve the structural integrity and functional properties of anthocyanins over extended periods, enhancing their stability during storage and processing. In addition, encapsulation helps to enhance bioavailability by protecting anthocyanin molecules from the harsh conditions of the gastrointestinal tract, allowing them to reach the small intestine intact, where absorption into the bloodstream occurs [29]. However, parameters used in the complexation method play a vital role in determining the morphology, structure, and antioxidant activity of anthocyanin–biopolymers complexes [29].

### 8.1. Acylation

Acylated anthocyanins offer better stability, bioavailability, and biological activities when compared with non-acylated anthocyanins. Acylated anthocyanins exist naturally in many plant species. Alternatively, they can also be formed by utilizing in vitro and in vivo methods. Four common methods have been suggested for the acylation of anthocyanin, including (1) in vivo biosynthesis by genetic modification of plants with genes encoding acyltransferases; (2) semi-biosynthesis, which combines in vivo biosynthesis and chemical synthesis; (3) chemical acylation; and (4) enzymatic acylation [28,41].

Lipase enzyme-mediated acylation of raspberry anthocyanins (cyanindin-3-*O*-glucoside) with methyl salicylate showed a conversion rate of 84.26% [66]. The acylation was on the glucoside C-6, and the product was cyanidin-3-(6-salicyloyl) glucoside, which showed improved stability in light, heat, and oxidation environments and improved oxygen-free radical absorptive capacity (ORAC) capacities, as well as enhanced DPPH and ABTS free radicals activities [66]. Acylation of blueberry anthocyanins with maleic acid prepared by solid-phase grafting method showed improved color stability during storage, compared with their native non-acylated form [92]. Moreover, in this study, maleic acid grafted anthocyanins retained the pH-color response characteristics, similar to their native non-acylated form, which suggested their use in the pH-color response indicator packing material.

Anthocyanin-rich fractions isolated from blackcurrant (*Ribes nigrum* L., *cv* Mortti), including delphinidin-3-*O*-rutinoside, cyanidin-3-*O*-rutinoside, delphinidin-3-*O*-glucoside, and cyanidin-3-*O*-glucoside, enzymatically acylated with lauric acid showed significantly higher thermostability and lipid peroxidation inhibition activities [93].

### 8.2. Protein and Polysaccharides-Binding Approaches 

The anthocyanin composite particles with proteins [94,95,96] and polysaccharides [26,27] are effective methods to improve the stability (pH, light, and temperature), bioaccessibility, bioavailability, and nutritional properties of anthocyanin. Moreover, anthocyanin–dietary protein interactions can also improve the structure, function, and nutritional properties of dietary proteins [97,98]. Therefore, utilizing the anthocyanin–dietary protein interaction in the production and development of food products is substantially useful.

The nature of protein and polysaccharide polymers used in complexions substantially influence the thermal stability of bounded anthocyanins. For instance, blueberry anthocyanins stabilized with xanthan gum showed superior thermostability compared to β-glucan and konjac glucomannan [26]. Among the water-soluble fraction (WSF-P) and chelator-soluble fraction (CSF-P) of blueberry pectin, CSF-P showed greater binding with anthocyanin, resulting in more effective in preventing the anthocyanin from degradation; also, it provided higher stability under gastrointestinal simulation [27].

The inter-molecular interactions of anthocyanins with proteins and polysaccharides are primarily noncovalent, including hydrogen bonding, electrostatic interactions, and van der Waals forces [26]. Nevertheless, in certain conditions, covalent bonding may occur between anthocyanins and nucleophilic groups present in amino acid residues of diverse proteins, resulting in modifications to the functionality of the aggregate particles formed by anthocyanin–protein interactions. These modifications can impact digestibility, transepithelial absorption, and antioxidant activities [99]. Moreover, covalently bound complexes lead to discoloration due to oxidation reactions and the formation of quinones [99]. It has been suggested that interactions between anthocyanins and proteins are predominantly noncovalent in an acidic environment, while covalent linkages are predominant in an alkaline environment [97].

Complexing polyphenols from wild blueberry (*Vaccinium angustifolium* Aiton; rich in delphinidin-glucoside) and muscadine grape (*Vitis rotundifolia*; rich in delphinidin-diglucoside) pomaces with rice–pea protein isolate blend showed a higher level of recovery, antioxidant, and anti-inflammatory bioactivity postdigestion [95]. In this study, the recovery index (% total phenolics present post-digestion) was 62% and 69% from muscadine grape and blueberry and protein–polyphenol particles, compared to 31% and 23% for the respective unmodified pomace extracts. Moreover, protein–polyphenol particle digests retained 1.5 to 2-fold higher antioxidant capacity than unmodified extract digests.

Soy protein isolates have been shown to protect the anthocyanins via hydrophobic interactions and static quenching [100]. Preheat treatment and binding of cyanidin-3-*O*-glucoside, the major anthocyanin of the black soybean seed coat, induced significant alterations in the secondary structure of soy protein isolate, including an increase in β-sheet and β-turn accompanied by a decrease in α-helix and random coil structure. Preheated soy protein at 121 °C showed a strong binding affinity towards cyanidin-3-*O*-glucoside, also effectively enhancing the thermal stability of the black soybean seed coat extract. This enhancement was demonstrated by a substantial 70% reduction in the degradation rate.

### 8.3. Co-Pigmentation

Co-pigmentation is the vital phenomenon of non-covalent interactions and the formation of stable complexes with other colorless pigments and organic acids. Co-pigmentation substantially impacts the color expression and stabilization of anthocyanins in plants as well as in solutions [101]. Co-pigments are compounds containing π-conjugated systems, which prefer engaging in π-π stacking interactions with anthocyanins. Such associations between co-pigments and anthocyanins protect these molecules by preventing water molecules from initiating nucleophilic attacks, thereby enhancing their overall stability [64].

Intermolecular and intramolecular and co-pigmentation, self-association, and metal complexation are the most commonly utilized co-pigmentation methods to increase anthocyanin stability [41,102]. Amino acids, organic acids, polysaccharides, flavonoids, and phenolics could be used for co-pigmentation with anthocyanins [64].

Co-pigments form stable complexes with anthocyanins through non-covalent interactions, including hydrogen bonds, van der Waals force, and electrostatic interaction. These interactions increase the absorption of pigments (hyperchromic effect) within the visible spectrum. Also, they generally cause a shift in the wavelength of maximum visible absorption (λmax) toward longer wavelengths (bathochromic effect) [101]. These effects contribute to darker colors and a more pronounced purple tone in wines [101]. Consequently, co-pigmentation holds significant relevance within the wine industry. In a study, intermolecular co-pigmentation between malvidin-3-*O*-glucoside and (−)-epicatechin showed the highest bathochromic of 13 nm with a hyperchromic shift of 142.46%, compared to other commonly occurring anthocyanin mono-glucosides and phenolics of red wine [101]. Similarly, in another study, the presence of chlorogenic acid as co-pigments, *Hibiscus sabdariffa* anthocyanins-rich extract showed a bathochromic effect of 6 nm [64].

The structures of pigments (e.g., hydroxylation and methoxylation pattern in B ring) and co-pigments determined the stability and color expression of co-pigmentation together [101]. In a study of co-pigmentation of mulberry juice anthocyanins, mainly composed of cyanidin-3-glucoside, the addition of kaempferol, hyperoside, rutin, quercetin, and isoquercitrin showed stronger binding affinity and thermostability compared to quercitrin and catechin [91].

### 8.4. Nano/Microencapsulation

The incorporation and delivery of anthocyanins in food products are limited due to their poor chemical instability. Nano/microencapsulation has been proven as the most appropriate way to minimize these limitations. Several methods have been investigated for the encapsulation of anthocyanins, including spray and freeze-drying, emulsification, gelation, and polyelectrolyte complexation [40,102,103]. Among them, spray-drying is most commonly utilized for microencapsulating anthocyanins, providing high encapsulation efficiency [40]. Carbohydrates, including gum Arabic, maize starch, and maltodextrin, as well as proteins such as gelatin, whey protein concentrate, and soy protein isolate, are extensively utilized as coating materials [40].

Characteristics of recently developed anthocyanins-based nano/microcapsules are listed in Table 2. It has been established that wall material substantially influences the physical properties of microcapsules, which impact anthocyanin degradation [104]. For instance, higher moisture contents of microcapsules cause the rapid degradation of anthocyanin [104]. Pieczykolan and Kurek [104] investigated micro-encapsulation of chokeberry using maltodextrin as a coating material with guar gum, pectin, β-glucan, inulin, and gum Arabic, as wall material. In this study, guar gum provided the lowest moisture contents (1.66%), highest encapsulation efficiency (92.98%), smallest particle size (16.29 µm) of microcapsules, and lowest degradation of 5.81% during 7 days of storage with the access of light and air. Gum Arabic provided the highest moisture contents (2.73%), lowest encapsulation efficiency (78.61%), largest particle size (53.09 µm) of microcapsules, and highest degradation of 20.42% during 7 days of storage.

Shaddel et al. [105] used a double emulsion (preparation of primary W/O emulsion and then double W/O/W emulsion) technique prior to complex coacervation using gelatin and gum Arabic to diminish the instability of black raspberry anthocyanins. The anthocyanin-loaded microcapsules showed increased storage stability up to 23.66% after 2 months of storage at 37 °C.

Anthocyanin extract of grape skin encapsulated in sodium alginate by emulsification/internal gelation followed by spray showed higher encapsulation efficiency, and light, thermal, and pH stability, compared to freeze-dried powders [106]. Moreover, in this study, under simulated gastric and intestinal digestion in vitro, during the last phase of intestinal digestion, freeze-dried powder showed the highest retention rate of 24.5%, while freeze-dried and free (non-capsulated) anthocyanin showed only 15 and 1% of retention efficiency, respectively.

Nanoparticles of anthocyanins extract of red raspberry pomace with β-lactoglobulin (β-Lg) prepared with desolvation combined with ultrasonication were more stable in the mouth (pH 6.8), simulated gastric (pH 2), and simulated intestinal (pH 6.9) by showing high retention rate than that of unencapsulated anthocyanins [107]. In this study, anthocyanin-loaded β-Lg nanoparticles showed higher (19.23%) bioavailability compared to unencapsulated anthocyanin (11.27%).

## 9. Bioaccessibility and Bioavailability

In nutrition research, bioaccessibility refers to the quantity of a nutritionally vital compound released from the food matrix and crosses membranes during its journey through the stomach and small intestine [42]. On the other hand, bioavailability encompasses the proportion of the nutritionally vital compound capable of being absorbed and available for use. According to the US Food and Drug Administration, bioavailability is defined as the “rate and extent to which an active ingredient or moiety is absorbed and becomes available at the site of action” [111].

The actual health benefits of bioactive compounds rely on their bioaccessibility and bioavailability in the human body. Animal and human clinical studies indicated that only ~1% of ingested anthocyanins are generally absorbed [39,42,44]. The absorption of anthocyanin largely depends on dose (low absorption from large doses), interindividual differences in the gut microbiota composition, and diet composition, i.e., the presence of other polyphenols, proteins, and fats [16,112,113,114]. Moreover, the presence of diverse hydroxyl groups, sugar moieties, and acylated groups significantly impacts the size, spatial conformations, and polarity of anthocyanin, which influence absorption [39,112]. For instance, pelargonidin (no substituents at C3′ and C5′ position of B-ring) derivatives are more readily absorbed than anthocyanins with more substituents on their B ring, such as peonidin (-OCH_3_ at C3′), delphinidin (OH- at C3′ and C5′), and cyanidin (-OH at C3′)-based anthocyanins [44]. Furthermore, among attached sugar moieties, malvidin 3-*O*-arabinoside showed higher bioavailability than malvidin 3-*O*-glucoside [44].

The gastrointestinal microbiota, including *Bifidobacterium* spp. and *Lactobacillus* spp., play a crucial role in the biotransformation and metabolism of anthocyanins, leading to changes in their chemical structure. As a result of this biotransformation, the intestine and colonic uptake of intact anthocyanins is reduced [115]. However, in recent years, encapsulation technologies, targeted site-specific delivery (e.g., colon and intestinal), and controlled release strategies have shown promising results in improving the stabilization of anthocyanin in the gastrointestinal tract to allow intact delivery for potentially enhancing bioavailability [42,95,97,103,107,116]. Moreover, acylation provides structural stability to anthocyanins, making them less prone to degradation or modification during digestion in the gastrointestinal tract; thus, acylated anthocyanins show higher bioavailability than non-acylated anthocyanins [47].

The small intestine is the major location of anthocyanin absorption [117]. Hydrophilic anthocyanin glycosides are hydrolyzed in the small intestine to less hydrophilic aglycones. Consequently, these aglycones can pass through the phospholipid bilayer membrane either through passive diffusion or with the assistance of specific transporters [117]. Recent studies revealed that anthocyanins can also be absorbed in the stomach [117,118]. In 12 human subjects (males and females with a mean age of 29.5 years), after the intake of a single dose of 1000 mg proprietary, standardized maqui berry (*Aristotelia chilensis*) extract containing 78 mg delphinidin-3-*O*-glucoside + 9.7 mg cyanidin-3-*O*-sambubioside, the maximum concentrations of delphinidin-3-*O*-glucoside and cyanidin-3-*O*-sambubioside in blood were observed after 1 and 2 h, respectively. Interestingly, in this study, some subjects had their individual maximum concentrations just after 0.5 h of intake, suggesting anthocyanin uptake in stomach, followed by continued reabsorption in the small intestines, and, to a certain extent, in the colon as well. Anthocyanins are absorbed in the stomach without undergoing deglycosylation, utilizing specific transporters such as sodium-dependent glucose co-transporter 1 and facilitative glucose transporters 1. However, in the small intestine, their primary mode of absorption is as aglycones [117]. The colon is the primary site of anthocyanin degradation, where the microbiota efficiently decompose anthocyanins into small, absorbable phenolic acids [117].

## 10. Health Benefits of Anthocyanins

The pre-clinical and clinical studies have suggested the beneficial effects of anthocyanin in minimizing the risk of cardiovascular diseases [10,45,119,120,121,122], diabetes [11], obesity [12], neurodegenerative diseases [13,123,124], cancer [14,125,126], and several other diseases associated with metabolic syndromes [15,127,128]. Primarily, these health benefits are modulated by the antioxidant activities of anthocyanins [1,15,16], as well as anthocyanin-mediated alternations in the gut microbiome [17,18,19,20,21]. It has been hypothesized that adding 12–150 mg of anthocyanin to the daily diet may provide these health benefits [127].

Recent research indicates that anthocyanin possesses prebiotic activity, which keeps the body healthy [20]. A recent review of 34 prebiotic activity studies of anthocyanins revealed that anthocyanins play a key role in promoting the proliferation of probiotics (e.g., *Bifidobacterium* and *Lactobacillus*), inhibiting the growth of harmful bacteria (e.g., *E. coli*, *Salmonella*, *S. aureus*), and improving the intestinal environment. In addition, anthocyanins promote the production of short-chain fatty acids (SCFAs, e.g., butyric, isobutyric, acetic, and propionic) and lactic acid [20].

Anthocyanin, as well as its metabolites produced by the actions of the gut microbiome, have shown potent bioactivity. For instance, protocatechuic acid is the main metabolite of cyanidin-3-glucoside, mainly produced by gut microbiota [129]. Anthocyanin-rich blueberry extracts and protocatechuic acid have shown beneficial effects in in vivo and in vitro models of Alzheimer’s disease [129]. In this study, APP/PS1 mice given 150 mg/kg BBE daily for 16 weeks decreased p62 protein levels, suggesting enhanced autophagosome degradation and alleviated neuron damage. Similarly, in this study, protocatechuic acid reversed the Aβ_25-35_-induced cytotoxicity in primary hippocampal neurons by promoting autophagosome degradation and reducing lactate dehydrogenase (LDH) and ROS levels.

It has been shown that acylated anthocyanins may have greater modulating effects on energy metabolism, inflammation, and gut microbiota in type 2 diabetes than nonacylated anthocyanins [130].

### 10.1. In Vitro Studies

The in vitro studies underscore the health-promoting benefits of anthocyanins (Table 3). Polyphenols, including anthocyanins, have shown prebiotic effects by altering the relative abundances of beneficial intestinal microbiota [63]. Blueberry anthocyanin, rich in malvidin-glucoside and malvidin-galactoside, showed an impact in increasing the relative abundances of some certain communities, especially *Bifidobacterium*, a widely recognized probiotic genus, effective in restoring intestinal homeostasis [63].

Anthocyanins-rich black currant fruit extract showed a greater effect on postprandial hyperglycemia by inhibiting α-glucosidase activity, compared to green currants (*Ribes nigrum* L.), which contains a low amount of anthocyanin [131]. However, both black current and green current are rich sources of polyphenolic compounds, which have been shown to potentially regulate the salivary α-amylase, 2-deoxy-D-glucose uptake, and glucose and fructose transporters in this study [131].

Diabetic retinopathy, a prevalent complication of type 2 diabetes, is a prominent factor leading to vision loss and blindness in individuals with diabetes. The main driving force behind the progression of diabetic retinopathy is elevated blood sugar levels (hyperglycemia) [132]. Blueberry extracts containing malvidin, malvidin-3-glucoside, and malvidin-3-galactoside, have attenuated the high glucose-induced cell cytotoxicity, expression of Nox4, intercellular adhesion molecule-1 (ICAM-1), nuclear factor-kappa B (NF-κB), and elevated NO and ROS levels in human retinal capillary endothelial cells (HRCECs) via antioxidant and anti-inflammatory mechanisms [132].

Glycation, which involves binding a sugar molecule to a protein, lipid, or nucleic acid, is linked to various neurodegenerative conditions, including Alzheimer’s disease. In Alzheimer’s disease, glycation enhances the aggregation and harmful effects of proteins like β-amyloid (Aβ). In a study, crude extracts of blackberry, blueberry, black raspberry, cranberry, strawberry, and red raspberry were fractionated to yield anthocyanins-free and anthocyanins-enriched extracts [133]. The berry anthocyanins (100 µg/mL) showed higher reactive carbonyl species trapping, free radical scavenging, and anti-glycation effects than their respective anthocyanins-free extracts. The berry anthocyanins (100 µg/mL) inhibited thermal and methylglyoxal-induced fibrillation of Aβ. Furthermore, at a concentration of 20 µg/mL, the anthocyanins present in berries attenuated H_2_O_2_-induced ROS production and decreased lipopolysaccharide (LPS)-induced nitric oxide species in BV-2 microglia. Additionally, they exhibited a decline in H_2_O_2_-induced cytotoxicity and caspase-3/7 activity in BV-2 microglia.

### 10.2. Animal Studies

Anthocyanins have demonstrated significant health advantages in animal studies (Table 4). It is well known that the gastrointestinal tract actively regulates overall body physiology, apart from its central role in food digestion. Following food consumption, anthocyanins and their metabolites accumulate substantially in the intestinal lumen; their physiological effects within the gastrointestinal tract account for local and systemic health benefits [136].

Anthocyanins–gut microbiota interactions are well known to modulate the therapeutic potential of anthocyanins against various chronic diseases [137]. Numerous current studies suggest that anthocyanins and their colonic metabolites have the potential to act as modifiers, influencing the gut microbiota by inhibiting the proliferation of harmful bacteria while fostering the growth of beneficial bacteria like *Lactobacillus* spp. and *Bifidobacterium* spp. [137,138]. Anthocyanins have the potential to alter the composition of gut microbiota, promoting a balanced micro-ecology. This, in turn, influences fat metabolism, gut epithelial function, biosynthesis of microbial SCFAs, communication with the central nervous system (CNS), and immune response [137]. Moreover, studies have suggested the role of anthocyanin in improving the intestinal barrier by improving the enhanced production of tight junction proteins associated with mucus production and cellular morphology, which reduce the risk of inflammation [138].

Research on compounds for clearing senescent cells (called “senolytics”) has shown potential in recent years. Owing to their anti-inflammatory and antioxidant activities, anthocyanins have shown “senolytics” effects in anti-aging [139]. Anthocyanins extracted from *Sambucus canadensis* fruits showed a significant reduction in cell senescence and the aging of the lens by suppressing the activity of the phosphoinositide 3-kinase (PI3K)/protein kinase B (AKT)/mammalian target of rapamycin (mTOR) signaling pathway. Consequently, anthocyanins attenuate aging by promoting the autophagic and mitophagic flux, apoptosis of senescent cells, and renewal of the mitochondria and the cell to maintain cellular homeostasis [139].

Obesity is widely recognized for its association with metainflammation, characterized by a prolonged inflammatory response that contributes to a range of metabolic disorders, often linked to elevated oxidative stress. Considering this, anthocyanins exhibit the potential to regulate multiple intracellular mechanisms, thereby reducing oxidative stress and mitigating metainflammation [140]. In C57BL/6J mice fed with a high-fat diet (HFD), anthocyanin from *Lycium ruthenicum* Murray fruits at doses of 50–200 mg/kg was administered for 12 weeks, which enriched SCFA-producing bacteria (e.g., *Akkermansia*, Ruminococcaceae, *Muribaculaceae*, and *Bacteroides*) SCFA content. Consequently, anthocyanin supplementation reduced weight gain, endotoxin-producing bacteria (e.g., Desulfovibrionaceae and *Helicobacter*), endotoxin (i.e., LPS) levels, and intestinal inflammation by inhibiting the LPS/NF-kB/Toll-like receptor 4(TLR4) pathway [141].

Hypermethylation of genes encoding secreted frizzled-related proteins (SFRPs) is associated with colorectal cancer [21]. The gut microbiota, immune system, and epigenetic modifications are key in methylation. Anthocyanin-rich black raspberry powder has shown colorectal cancer chemopreventive effects in azoxymethane/dextran sodium sulfate (DSS)-treated C57BL/6J mice by modulating the gut microbiota and changes in inflammation and the methylation status of SFRP2 [21]. In this study, supplementation of raspberry powder (10 mg/kg body weight) reversed the imbalance in gut microbiota by decreasing the *Enterococcus* sp. and *Desulfovibrio* sp. and increasing the probiotics, including *Eubacterium rectale*, *Lactobacillus*, and *Faecalibacterium prausnitzii*. Moreover, the expression levels of DNA methyltransferase (DNMT)31 and DNMT3B and signal transducer and activator of transcription-3 (p-STAT3) were downregulated in animals supplemented with anthocyanin-rich black raspberry powder.

In a systematic review of 12 pre-clinical studies, anthocyanin was found to be beneficial for Alzheimer’s disease by minimizing oxidative stress, reactive astrogliosis, neuroinflammation, apoptosis, neuronal extracellular calcium, cholinergic dysfunction, synaptotoxicity, tau hyperphosphorylation, dysregulated membrane potential, and dysfunctional amyloidogenic pathway [142].

**Table 4:** Health benefits of anthocyanin evaluated in animal models.

Source of Anthocyanin and Dose	Experimental System	Bioactivity/Disease	Significant Results	Reference
Black raspberry powder, 10 mg/kg/day for 12 weeks	Azoxymethane/DSS-treated C57BL/6J mice	Colorectal cancer	↓ *Enterococcus* sp. and *Desulfovibrio* sp., ↑ probiotics (*Eubacterium rectale*, *Lactobacillus*, and *Faecalibacterium prausnitzii*), ↓ inflammatory cytokines (TNF-α and IL-6), ↑ expression of SFRP2 mRNA and protein, ↓ expression of DNMT31, DNMT3B, and p-STAT3	[21]
Blueberry extract, 150 mg/kg daily for 16 weeks	double-transgenic APP/PS1 mice	Alzheimer’s disease	↓ Neuron damage, ↑ degradation of autophagosomes (↓ p62 proteins), and neuronal autophagy (↑LAMP1 and cathepsin D proteins)	[129]
Fruits of *Sambucus canadensis*, 50–200 mg/kg/day for 8 weeks	male Kunming mice	Anti-aging	↓ Activity of PI3K/AKT/mTOR pathway to promote autophagic and mitophagic flux in senescent cells ↑ Clearing damaged mitochondria and apoptosis of senescent cells to attenuate aging	[139]
*Lycium ruthenicum*, 50–200 mg/kg for 12 weeks	C57BL/6J mice fed with HFD	Obesity	↓ Weight gain, enriched SCFA-producing bacteria (e.g., Akkermansia, Ruminococcaceae, Muribaculaceae, and Bacteroides), SCFA content, endotoxin-producing bacteria (e.g., Desulfovibrionaceae and Helicobacter), endotoxin (i.e., LPS) levels, and intestinal inflammation by inhibiting the LPS/NF-κB/TLR4 pathway	[141]
Roselle (*Hibiscus sabdariffa* L.) extract, 0–400 mg/kg for 35 days	chicks (Ross 308 broiler)	Normal growth and development	↑ n-3 PUFA in the breast muscle, immunoexpression of IgG in the spleen, serum thyroxine hormone (T4), serum SOD level, serum levels of lysozymes and IL10, serum complement 3 (C3) level, ↓ serum triglyceride, serum MDA	[143]
*Lycium ruthenicum* Murray, 200 mg/kg/day or 3 months	male C57BL/6 mice	Normal growth and development	↑ Liver T-AOC, CAT, T-SOD, GSH, and GSH-Px, ↓ liver AST, ALP, ALT, and MDA, ↑ anti-inflammatory status in colon (↓ expression of mRNA of Cox-2, iNos, TNF-α, IL-6, IL-1β, and Ifn-γ), improved intestinal barrier (↑ expression of mRNA of Muc1, Zo-1, Occludin, and Claudin-1), ↑ beneficial gut microbiota (*Barnesiella*, *Alistipes*, *Eisenbergiella*, *Coprobacter* and *Odoribacter*), ↑ SCFA in cecal contents and feces	[144]
*Vaccinium uliginosum* extract	hairless mice (SKH-1)	Skin aging in UVB-induced photodamage	↓ Expression of MMP mRNA, ↑ mRNA expression of TIMP, and antioxidant-related genes (SOD1, CAT, and GPx), ↓ protein levels of inflammatory cytokine (IL-6, IL-12, and TNF-α), ER, JNK, and p38	[145]
*Lycium ruthenicum* Murray fruit extract, 20 mg/kg/day	male C57BL/6 mice	Ulcerative colitis and Crohn’s disease	↓ Proinflammatory cytokines mRNA (TNF-α, IL-6, IL-1β, and IFN-γ), promotion of the intestinal barrier function (↑ ZO-1, occluding, and claudin-1 proteins), gut microbiota modulation (↑ *Lactobacillus*, ↓ *Oscillibacter*)	[146]
Bilberry extract, 10–40 mg/kg/day for 10 weeks	female SD rats	Anti-aging	↑ Beneficial intestinal bacteria (*Aspergillus oryzae*, *Bacteroides*, *Lactobacillus*, *Clostridiaceae-1*, the *Bacteroidales-S24-7-group* and the *Lachnospiraceae_NK4A136_group*), ↓ harmful bacteria (*Euryarchaeota and Verrucomicrobia*), ↑ SCFAs (acetic acid, butyric acid, and propionic acid) ↓ activity of digestive enzymes (α-glucosidase, α-galactosidase, β-galactosidase, β-glucosidase, and β-glucuronidase), ↓ colon TNF-α and IL-6	[147]
Korean black beans, 24 mg/kg/day for 2 weeks	LPS-induced neurotoxicity in Male C57BL/6N mice	Alzheimer’s disease	↓ Inflammatory markers (p-NF-kB, TNF-α, and IL-1β), neuronal apoptosis (↓ expression of Bax, cytochrome c, cleaved caspase-3, and cleaved PARP-1), ↑ the level of survival proteins p-Akt, p-GSK3β, and antiapoptotic Bcl-2 protein.	[148]

Increase and decrease are symbolized by upward (↑) and downward (↓) arrows. Abbreviations are as follows. AKT: protein kinase B; ALP: alkaline phosphatase; ALT: alanine aminotransferase; AST: alanine aminotransferase; CAT: Catalase; Cox-2: yclooxygenase-2; DNMT: DNA methyltransferase; DSS: dextran sodium sulfate; GSH: reduced glutathione; GSH-Px: glutathione peroxidase; HFD: high-fat diet; Ifn-γ: interferon γ; IgG: Immunoglobulin G; IL: interleukin; iNos: inducible nitric oxide synthase; JNK: Jun N-terminal kinase; LAMP1: lysosomal-associated membrane protein 1; LPS: lipopolysaccharide; MDA: malondialdehyde; MMP matrix metalloproteinase; mTOR: mammalian target of rapamycin; Muc1: mucin-1; NF-κB: nuclear factor kappa-light-chain-enhancer of activated B cells; PI3K: phosphoinositide 3-kinase; p-STAT3: signal transducer and activator of transcription-3; PUFA: polyunsaturated fatty acids; SCFA: short-chain fatty acids; SFRP2: secreted frizzled-related protein 2; SOD: superoxide dismutase; T-AOC: total antioxidant capacity; TIMP: tissue inhibitor of metalloproteinase; TLR4: Toll-like receptor 4; TNF-α: tumor necrosis factor α; T-SOD: total superoxide dismutase; Zo-1: zonulae occludens-1.

### 10.3. Clinical Trials

Similar to the in vitro and animal studies, case–control, cohort, and interventional studies have also demonstrated the health benefits of bioactive compounds derived from citrus fruits.

A meta-analysis of 21 clinical trials and 27 pre-clinical studies observed a dose-dependent effect of dietary anthocyanins intake on biomarkers of metabolic syndrome, including type 2 diabetes (hyperglycemia) and associated obesity [43]. The meta-analysis found that daily anthocyanin intake of more than 5 mg/kg of body weight significantly reduced fasting blood glucose, glycosylated hemoglobin (HbA1c), total cholesterol, and triglycerides and increased high-density lipoprotein (HDL) levels. Moreover, anthocyanin-rich food significantly reduced low-density lipoprotein (LDL) levels in clinical trials.

A systematic review meta-analysis of 8 clinical trials consisting of 715 patients (165 men and 195 women) with chronic kidney disease undergoing hemodialysis showed that anthocyanins-rich intervention reduced inflammatory parameters, oxidative stress, and improved lipid profile [149]. In this meta-analysis, anthocyanin intervention decreased the oxidant parameters, and especially reduced malondialdehyde products and myeloperoxidase, against the placebo group. Moreover, anthocyanin intervention increased HDL cholesterol levels.

A meta-analysis of 39 human clinical trials revealed that anthocyanin intake promotes functional and subjective recovery after exercise [150]. Anthocyanin intakes showed beneficial effects by reducing creatine kinase, muscle soreness, strength loss, and improving power after exercise. This was accompanied by attenuated C reactive protein and TNF-α.

A meta-analysis comprising 9 prospective cohort studies on a total of 602,054 participants, with over 22,673 reported cases of non-fatal or fatal cardiovascular disease, it was found that consuming dietary anthocyanins was associated with a lower risk of coronary heart disease (relative risk (RR) = 0.91) and cardiovascular disease-related mortality (RR = 0.92). However, no significant correlation was observed between the intake of these compounds and a reduced risk of myocardial infarction (MI), stroke, or overall cardiovascular disease [121].

In a randomized placebo-controlled cross-over study on 25 healthy female and male adults (19–35 years), supplementation of anthocyanin-rich extract showed beneficial effects against unhealthy 1026 kcal high-fat meal by modulating the associated endotoxemia, alterations of glycemia and lipidemia, postprandial dysmetabolism, and redox and insulin signaling [151]. In this study, the supplementation of extract containing 320.4 mg of anthocyanins (166.4 mg cyanidin and 121.7 delphinidin) alleviated the high-fat meal-induced postprandial dysmetabolism, including (i) increases in plasma lipopolysaccharides (LPS) and LPS-binding protein (endotoxemia); (ii) plasma glucose and triglyceride increases; (iii) oxidative stress biomarkers -TNF-α and NADPH oxidase 4 (NOX4) upregulation in peripheral blood mononuclear cells (PBMC); and (iv) insulin signaling biomarkers c-Jun N-terminal kinases (JNK1/2) activation in PBMC. However, in this study, anthocyanin did not significantly affect high-fat meal-induced-mediated increases in plasma insulin, gut-secreted hormones glucagon-like peptide-1 (GLP-1), GLP-2, and gastric inhibitory polypeptide (GIP) involved in the regulation of insulin secretion, and LDL- and HDL-cholesterol, and oxidative stress biomarker IκB kinase (IKK) phosphorylation in PBMC.

In a randomized controlled trial on 176 dyslipidemia subjects (35–70 years), anthocyanin supplementation of 80–320 mg/d for 12 weeks improved serum HDL-cholesterol levels, HDL-induced cholesterol efflux capacity (CEC), and apolipoprotein A-I (APOA-I) in a dose–response manner [152]. Moreover, in this study, the enhancement of CEC showed positive correlations with the increase in HDL-C and APOA-I. Interestingly, the positive effect of anthocyanin on CEC and lipid profile was not visible within 6 weeks of supplementation, suggesting that the duration of anthocyanin supplementation is a vital factor for improving lipid metabolism.

In another randomized controlled trial by the same group of researchers on 169 dyslipidemia subjects, anthocyanin supplementation of 40–320 mg/d for 12 weeks showed dose-dependent effects on inflammatory cytokines and oxidative stress biomarkers [153]. In this study, the highest benefits were observed in the highest dose (320 mg/d) group (n = 42), which showed the highest decrease in serum interleukin (IL)-6 (−40%), TNF-α (−21%), malonaldehyde (MDA; −20%), urine 8-iso-8-isoprostaglandin (PG) F2α (−37%), and -hydroxy-2′-deoxyguanosine (8-OHdG; −36%), compared to placebo group.

In randomized controlled trials, anthocyanin metabolites of blueberries are identified as major mediators of beneficial vascular functions and changes in cellular gene programs involving immune response, cell adhesion, migration, and cell differentiation [46]. Daily 1-month wild blueberry consumption, containing 150 mg anthocyanins, increased flow-mediated dilation (FMD; 2.1%) and lowered 24 h ambulatory systolic blood pressure (−5.6 mmHg). Moreover, out of the 63 anthocyanin plasma metabolites quantified in this study, 21 and 14 correlated with chronic and acute FMD improvements, respectively. Interestingly, injection of these metabolites into male C57BL/6 mice improved FMD.

## 11. Industrial Applications of Anthocyanins

The anthocyanin market was estimated to be worth USD 348.7 million in 2022, which is expected to reach USD 348.7 million in the year 2032 with a compound annual growth rate of 4.3% [154]. The intensive use of anthocyanins as natural colorants in various food and drink formulations, as well as bioactive compounds in nutraceuticals, pharmaceuticals, specialty drugs, and premium beauty and personal care products, has extensively boosted their market requirement [154]. Moreover, using anthocyanin as a natural source for smart and intelligent packaging has fueled the demand for anthocyanins.

### 11.1. Food Colorant

The EFSA has approved the use of anthocyanins-rich aqueous extracts as food dyes (E 163), acknowledging their safety for consumption in food products [14,30]. This approval highlights the potential of anthocyanins as a natural food colorant. Anthocyanins play a pivotal role as primary quality indicators in red wines, serving as the predominant source of the red color in red grapes and the resulting wines [155]. Moreover, anthocyanins are extensively used as natural colorants in diverse foods, including confectioneries, preserves such as jams and jellies, and sausages. They are also prominently utilized in several beverages, ranging from dairy products like yogurts to fruit juices. This widespread utilization underscores the versatility and significance of anthocyanins in the food and beverage industry.

The addition of anthocyanins in food products substantially improves the antioxidant, antimicrobial, and prebiotic activities. Anthocyanin-rich extract prepared from Jabuticaba (*Myrciaria jaboticaba* (Vell.) O. Berg.) fruits epicarp utilized in Macarons as a coloring agent showed more stable color than the commercial colorant E163 during a 6-day shelf-life [156].

At the industrial production scale, anthocyanin pigments are largely produced by extraction from waste grape skins from the wine industry, red cabbage, black carrots, sweet potato, and berries [89]. However, in the future, microbial and plant cell cultures may be utilized in the commercial production of anthocyanins [88,89].

### 11.2. Anthocyanins-Based Active and Intelligent Packaging

Active and intelligent packaging refers to new innovative packaging systems designed to interact with the food product or its environment to provide improved shelf-life and/or real-time monitoring of food freshness [52]. As microbial growth and oxidation are the main causes of food deterioration, anthocyanin-based active packaging films prepared with the complexion of anthocyanins with biopolymer exhibit excellent antioxidant and antimicrobial properties, thus minimizing food deterioration [157,158,159].

**Figure 4:**
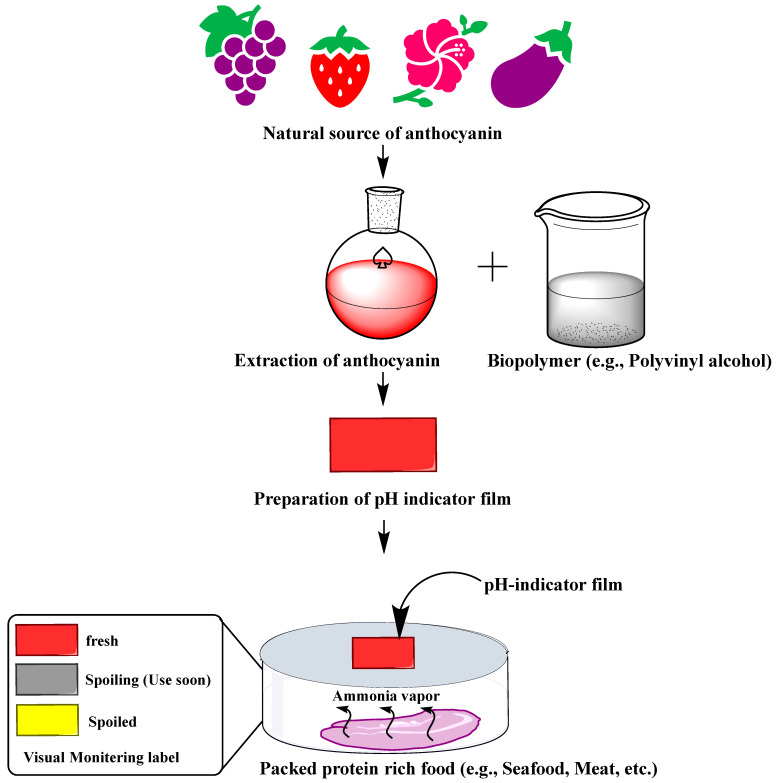
Process of preparation of anthocyanin-based pH-indicator film and their use in the freshness monitoring of protein-rich foods.

Microbial growth and metabolism during the spoilage of protein-rich food (e.g., seafood) produce volatile nitrogen compounds such as ammonia (NH3), trimethylamine (TMA), and dimethylamine (DMA), which create alkaline conditions (an increase in hydroxyl ions) [160]. Consequently, because of the pH-sensitive properties of anthocyanins, the pH changes are sensed by anthocyanin-based films and change their color, enabling visual monitoring of the freshness of protein-rich food [35,36] (Figure 4). For instance, barberry anthocyanins incorporated chitin nanofiber and methylcellulose composite film showed a color change from reddish to pale pink with an increase in pH values of the fish sample from 6.3 to ~8.0 during the 72 h storage [161]. Similarly, red cabbage anthocyanin-agarose film is capable of discriminating between fresh (pH 6.8), spoiling (pH 5.5), and spoiled (pH 4.0) milk [162]. The sensor undergoes a visible blue (pH 6.8) to purple (pH 5.5) to pink (pH 4.0) color change in response to lactic acid accumulation (an indicator of microbial spoilage of milk) [162].

Anthocyanins-based composite films are widely used to extend the shelf-life of food (e.g., cheese, milk, and fruits) and in real-time monitoring of freshness of protein-rich food products, including aquatic (e.g., fish and shrimp), meat (e.g., chicken, beef, and pork), and dairy products (e.g., milk, yogurt, and cheese) [31,32,33,34]. This provides a must-required alternative to traditionally used synthetic non-edible and non-biodegradable polymers.

In developing anthocyanin-based composite film, polysaccharide-based (e.g., chitosan, cellulose, and starch) or protein-based (e.g., gelatin, zein, and soybean protein isolate) are the biopolymers widely utilized (Table 5).

Rawdkuen et al. [163] tested anthocyanin-rich extracts of different plants, including sweet potatoes, red cabbage, roselle (*Hibiscus sabdariffa*), butterfly pea (*Clitoria ternatea*), husks and peelings from mangosteen (*Garcinia mangostana*), and red dragon fruit (*Hylocereus undatus*), to develop intelligent gelatin films. Among them, anthocyanin extracted from butterfly pea showed the highest pH sensitivity by displaying distinct color changes at each pH, suggesting that butterfly pea anthocyanin extracts are a promising candidate for formulating smart/active films for food freshness monitoring.

Green halochromic smart and active packaging materials were developed using TiO_2_ nanoparticles and red barberry anthocyanins anthocyanin-loaded gelatin/κ-carrageenan films and utilized to monitor fish freshness [164]. This study demonstrated that the addition of TiO_2_ nanoparticles and natural anthocyanins resulted in a substantial enhancement of the films’ mechanical strength and moisture resistance. The film exhibited color changes corresponding to variations in the freshness of the fish samples, which showed a correlation with ammonia production during the degradation of the fish. Furthermore, the inclusion of anthocyanins and TiO_2_ nanoparticles augmented the bacteriostatic and antioxidant properties of the intelligent films.

Active and intelligent packaging films with antioxidant, antibacterial, and colorimetric pH indicator properties were developed by incorporating *Clitoria ternatea* extract into gellan gum film and utilized for the freshness monitoring of shrimp [165]. In this study, gellan gum film blended with heat-treated soy protein isolate (HSPI) substantially reduced the swelling capacity, water vapor permeability, hydrophobicity, and tensile strength of the composite film and controlled the anthocyanins release at pH greater than 6.0. As the shrimp underwent progressive spoilage, the intelligent films underwent color changes in correlation with the increase in total volatile basic nitrogen (TVBN) values.

In addition to intelligent/smart packaging film, red cabbage anthocyanin immobilized carboxymethyl cellulose/polyvinyl alcohol composite films have been developed to supervise the condition of wound healing (onsite wound pH measurement) [166]. In this study, the dyed samples displayed a color change from purple to pink after spraying with a wound mimic solution with a pH value below 7, suggesting that this colorimetric assay can be used for wound healing monitoring.

**Table 5:** Anthocyanins-based active and intelligent packaging films and their application.

Source of Anthocyanin	Biopolymer	Application/Functional Properties	References
Rice bran	Chitosan matrix embedding oregano essential oil	Preserving pork and monitoring freshness, decreases the abundance of spoilage bacteria related to stress tolerance, pathogenicity, and biofilm formation in the pork.	[157]
Purple sweet potato and red cabbage extracts	Corn starch and polyvinyl alcohol	Purple sweet potato film exhibited bolder color, better mechanical properties, and lower light transmittance than film prepared with red cabbage extracts. Visual color changes corresponding to Volatile basic nitrogen (TVB-N) variation when shrimps were spoiled.	[160]
Red barberry	Methylcellulose with chitin nanofiber	The indicator transitions from a reddish/crimson color to pink to yellow as the pH increases and from pink to yellow as the concentration of ammonia vapor increases. Monitoring of fish freshness.	[161]
Red barberry	Gelatin and κ-carrageenan, with TiO_2_ nanoparticles	Incorporation of TiO_2_ nanoparticles and anthocyanins substantially improved the mechanical and moisture resistance of the films. Monitoring of fish freshness.	[164]
*Clitoria ternatea* extract	Gellan gum with heat-treated soy protein isolate (HSPI)	Antioxidant, antibacterial, and colorimetric pH indicator. HSPI substantially reduced the swelling capacity, water vapor permeability, hydrophobicity, and tensile strength of the composite film and controlled the anthocyanins release. Changed color with the increase in TVB-N values during progressive spoilage of shrimp.	[165]
Blood orange	Chitosan/gum Arabic	Increase shelf-life of milk, visually monitored freshness through color changes.	[167]
Red cabbage	Polyvinyl alcohol/sodium carboxymethyl cellulose	Real-time monitoring of pork freshness, antibacterial activity against *Escherichia* *coli* and *Staphylococcus aureus*.	[168]
Red cabbage	Sodium alginate, pectin, and cellulose nanocrystals	Monitoring the freshness of shrimp in real-time.	[169]
Torch ginger (*Etlingera elatior* (Jack) R.M.Sm.) extract	Sago starch	As the pH increased from 4 to 9, the color of the films transitioned from pink to a faint shade of green.	[170]
*Echium amoenum* flower extract	Bacterial cellulose	Monitoring of shrimp freshness following the total viable count (TVC) and TVB-N values, film color changed violet (fresh) → gray (spoiling, use soon) → yellow (spoiled)	[171]

## 12. Conclusions and Future Prospects

Anthocyanins are fascinating molecules with significantly diverse chemical structures and biological activities. Ample opportunities and directions exist for future research on these redox-active and nutritionally vital phytochemicals.

Antioxidant properties and gut microbiota-modulating properties make anthocyanins promising candidates for minimizing the risk of cardiovascular diseases, diabetes, obesity, neurodegenerative diseases, cancer, and several other diseases associated with metabolic syndromes. Anthocyanins play a key role in promoting the proliferation of probiotics (e.g., *Bifidobacterium* and *Lactobacillus*), inhibiting the growth of harmful bacteria (e.g., *E. coli*, *Salmonella*, and *S. aureus*), increase the production of SCFAs, and improving the intestinal environment. Moreover, anthocyanins are well-studied to upregulate antioxidant enzymes (e.g., SOD, CAT, and GPx) and decrease proinflammatory cytokine production.

Berries are the primary source of anthocyanin because they contain the highest amount of anthocyanin. While red wine, vegetables, and other fruits contribute 22–18, 19, and 9% of total anthocyanin. A significantly different level of anthocyanins has been reported in fruits and vegetables. Accurately quantifying anthocyanins while conducting appropriate quality control experiments is essential for effectively monitoring anthocyanin intake.

Anthocyanins undergo degradation due to diverse external environmental factors, thereby constraining their suitability for applications within the food industry. However, strategies have been developed to enhance their thermal and storage stability, which include modifying their chemical structure (acylation), combining them with proteins and polysaccharides to form stable complexes, and micro-/nano-encapsulation. These strategies have shown improved solubility, bioaccessibility, and bioavailability of anthocyanins. Especially, micro-/nano-encapsulation by spray-drying is widely investigated to enhance the shelf-life and storage stability of anthocyanins. Moreover, encapsulation protects these sensitive pigments during digestion and facilitates the controlled release and intact delivery to the colonic microbiota. However, encapsulation methods are not widely used in food industries due to their high production cost.

Pre-clinical (in vitro and in vivo) studies have shown the beneficial effects of anthocyanins in minimizing the incidence of several diseases associated with metabolic syndromes, especially type 2 diabetes and associated obesity, and neurodegenerative diseases. However, these experiments are not able to completely mimic the human physiology. Moreover, the role of anthocyanin-derived metabolites (catabolized at the gut microbiome), which enter circulation as phenolic metabolites, are not widely investigated. Thus, it is important to evaluate the health benefits of anthocyanins and their metabolites using human clinical studies with larger participants.

The pH-sensitive properties of anthocyanins are now widely investigated for developing active and intelligent packaging films. However, some issues should still be overcome before the successful implementation of anthocyanins-based indicators. Namely, the ability of anthocyanins to show distinct color changes at minor pH changes needs further investigation. For this, anthocyanins from diverse sources can be investigated comparatively.

Considering the numerous health benefits associated with consuming 12–150 mg/day of anthocyanin, incorporating anthocyanin-rich fruit, vegetables, and grains into the diet should be promoted.

Finally, we propose that future studies concentrate more on anthocyanin stabilization (in a cost-effective way) for improved stability and bioavailability and explore their health benefits using human clinical studies with larger numbers of participants.

## Figures and Tables

**Table 2:** Characteristics of anthocyanins-based nano/microcapsules.

Encapsulation Method	Core Material	Wall Material	Capsule Size (µm)	Encapsulation Efficiency (%)	Remarks	Reference
Spray-drying	Chokeberry	Maltodextrin + Guar gum, gum Arabic, pectin, β-glucan, or inulin	16.29 (guar gum)–53.9 (gum Arabic)	78.6 (gum Arabic)–92.98 (guar gum)	Maltodextrin + guar gum microcapsules provided the highest protection during 7 days of storage. Water contents of microcapsules directly associated with degradation rate of anthocyanins during storage.	[104]
Double emulsion + complex coacervation	Black raspberry fruit extract	Gelatin and gum Arabic	35.34–80.22	29.67–38.54	Increased storage stability up to 23.66% after 2 months of storage at 37 °C.	[105]
Emulsification/internal gelation followed by spray/freeze-drying	Grape skin extract	Sodium alginate	0.56 (spray-drying); 99.8 (freeze-drying)	75.12 (spray-drying); 70.07 (freeze-drying)	Spray-dried powders exhibited higher encapsulation efficiency and light, thermal, and pH stability than freeze-dried powders.	[106]
Desolvation combined with ultrasonication	Red raspberry pomace	β-lactoglobulin	0.129–0.351	77	Anthocyanin-loaded β-Lg nanoparticles showed higher (19.23%) bioavailability compared to unencapsulated anthocyanin (11.27%).	[107]
Aqueous two-phase system-based encapsulation	Pure anthocyanins	Collagen + pectin/chitosan	-	92.58	All-aqueous template for loading hydrophilic bioactive anthocyanins.	[108]
Freeze-drying	Red raspberry	Soy protein isolate and gum Arabic	21.07–48.19	93.05–98.87%	Improved thermal stability in the temperature range of 80–114 °C, Improved anthocyanin retention (up to 48%) during storage at 37 °C for 60 days, improved anthocyanin delivery, and bioavailability during simulated gastrointestinal conditions.	[65]
Freeze-drying	Blueberry	Carboxymethyl starch (CMS)/xanthan gum (XG)	-	96.51–97.16	CMS/XG ratio of 30/1 provided superior antioxidant stability of microcapsules. Anthocyanin stability increased by 76.11% after the 30-day storage at 37 °C.	[109]
Ionic gelation utilizing dripping-extrusion and atomization	*Hibiscus sabdariffa* L. calyces	Rapeseed oil/pectin	78–1100	67.9–93.9	The half-life (t_1/2_) of the microencapsulated anthocyanins ranged from 7 (25 °C) to 180 days (5 °C).	[110]

**Table 3:** Health benefits of anthocyanin observed using in vitro experimental systems.

Source of Anthocyanin and Dose	Experimental System	Bioactivity/Disease	Significant Results	Reference
Blueberry extract and its metabolite protocatechuic acid, 50–400 µM	Aβ_25-35_-induced cytotoxicity in primary hippocampal neurons	Alzheimer’s disease	↑ Neuron viability, ↓ levels of lactate dehydrogenase and reactive oxygen species, ↑ degradation of autophagosomes and neuronal autophagy	[129]
Blueberry, purified anthocyanin, 10.0 g/L	Anaerobic fermentation in vitro	Prebiotic	↑ Relative abundances of beneficial intestinal microbiota, including *Bifidobacterium* spp.	[63]
Black currants extract, 66 to 0.06 μg/mL	Human CaCo-2 cells	Type 2 diabetes	↓ α-glucosidase activity	[131]
Blueberry, Mv, Mv-3-glc, and Mv-3-gal; 10 μg/mL	HRCECs	Type 2 diabetes	↓ ROS, NO, ↑ CAT and SOD activity, ↓ high glucose-induced Nox4 expression, ↓ VEGF level and inhibiting Akt pathway, high glucose-induced ICAM-1 and NF-κB	[132]
Barry anthocyanins-enriched extracts, 20–100 μg/mL	Murine BV-2 microglia	Neurodegenerative disorders	↓ LPS-induced nitric oxide species, H_2_O_2_-induced cytotoxicity, and caspase-3/7 activity	[133]
Black lentil, blue wheat, sorghum, black peanut, black rice, and black and purple bean extracts	Human colon cancer cells, HCT-116 and HT-29	Colon cancer	↓ Expression of anti-apoptotic proteins, survivin, and cellular inhibitor of apoptosis 2 (cIAP-2/XIAP), ↑ apoptosis, and arrested cells in G1, highest inhibition of cancer cells by black lentil, sorghum, and red grape	[134]
Bilberry (*Vaccinium myrtillus* L.) fruit extract	α-glucosidase and α-amylase	Type 2 diabetes	Inhibited α-glucosidase (IC_50_ of 0.31 mg/mL) and α-amylase (IC_50_ of 4.06 mg/mL)	[135]

Increase and decrease are symbolized by upward (↑) and downward (↓) arrows. Abbreviations are as follows. CAT: catalase; HRCECs: human retinal capillary endothelial cells; ICAM-1: intercellular adhesion molecule-1; Mv: malvidin; Mv-3-gal: malvidin-3-galactoside (Mv-3-gal); Mv-3-glc: malvidin-3-glucoside; NF-κB: nuclear factor-kappa B; NO: nitric oxide; SOD: superoxide dismutase; VEGF: vascular endothelial cell growth factor.

## Data Availability

No new data were created or analyzed in this study. Data sharing is not applicable to this article.
